# Association between South African high-school learners’ knowledge about tuberculosis and their intention to seek healthcare

**DOI:** 10.3402/gha.v6i0.21699

**Published:** 2013-10-03

**Authors:** Saloshni Naidoo, Myra Taylor

**Affiliations:** 1 Discipline of Occupational and Environmental Health, School of Nursing and Public Health, University of KwaZulu-Natal, Durban, South Africa; 2 Discipline of Public Health, School of Nursing and Public Health, University of KwaZulu-Natal, Durban, South Africa

**Keywords:** high-school learners, tuberculosis, intention to seek treatment

## Abstract

**Background:**

South Africa has one of the highest prevalence of tuberculosis (TB). Addressing awareness among school learners on TB transmission and prevention may assist in reducing the disease burden.

**Objective:**

We report on factors associated with high-school learners’ intentions to seek healthcare for TB.

**Design:**

A survey testing TB symptoms, transmission, prevention knowledge, and intention to seek and adhere to treatment was conducted among 1,114 high-school learners in KwaZulu-Natal (KZN), South Africa. Multivariate models correcting for nesting of students within schools tested associations between demographics, TB symptoms, transmission, prevention knowledge, and intention to seek and adhere to treatment.

**Results:**

Learners knowing that coughing for more than 3 weeks (OR: 2.33; 95% CI: 1.35–4.00) and night sweats (OR: 3.12; 95% CI: 1.80–5.41) were TB symptoms, TB is transmitted when a person with TB coughs (OR: 1.56; 95% CI: 1.23–1.98), and coughing in a closed room was an incorrect practice for someone with TB (OR: 1.71; 95% CI: 1.05–2.78) were significantly more likely to intend taking family members for treatment. Learners knowing that coughing for more than 3 weeks (OR: 2.69; 95% CI: 1.19–6.09), coughing blood (OR: 2.24; 95% CI: 1.33–3.76), and night sweats (OR: 2.25; 95% CI: 1.09–4.64) were TB symptoms, were significantly more likely to intend encouraging family members to adhere to TB treatment. Learners knowing that coughing for more than 3 weeks (OR: 1.47; 95% CI: 1.05–2.07), coughing blood (OR: 2.08; 95% CI: 1.44–3.01), and weight loss (OR: 1.85; 95% CI: 1.38–2.49) were TB symptoms, were significantly more likely to intend taking TB treatment if symptomatic. Learners knowing that coughing for more than 3 weeks (OR: 2.04; 95% CI: 1.45–2.87), and coughing blood (OR: 1.81; 95% CI: 1.24–2.62), were TB symptoms were significantly more likely to intend adhering to TB treatment.

**Conclusions:**

High-school learners with knowledge about TB symptoms, transmission, and prevention have positive intentions to seek treatment for themselves and family members and adhere to treatment.

In South Africa, there has been a resurgence of tuberculosis (TB) as a result of the human immunodeficiency virus (HIV) being pandemic, and this country is thus one of the countries with the highest incidence of TB (1). Many patients fail to adhere to treatment and this has resulted in a high prevalence of multidrug-resistant (MDR) and extensively drug-resistant (XDR) TB, requiring new efforts to improve control of infection, enhance TB screening, and optimise TB treatment (2). TB is preventable but early diagnosis and treatment is required to reduce transmission. For this to occur, it is critical that TB symptoms are identified early and that patients attend health facilities where TB diagnosis and treatment is free, are tested, and subsequently receive and adhere to treatment (3). Within the population, a much greater awareness about TB transmission, symptoms, prevention, health-seeking behaviour, and adherence to treatment is required.

Studies have estimated the prevalence of latent TB infection in adolescents in South Africa to be more than 50% (4, 5). The high latent TB infection prevalence places this age group at risk of active TB infection. Since 1996, as a result of South Africa's compulsory education policy, many children attend school and the high enrollment makes the school an appropriate place for increasing awareness in the community and providing targeted information about TB (6). Children of high-school age with health literacy seek treatment (7) and thus can also explain to other family members about the benefits of seeking treatment. Learners who are aware of TB symptoms and what is required to prevent transmission (including hygienic practices and the importance of ventilation) (8) and promote treatment can seek testing and treatment for themselves and also encourage family members to be tested for TB and to adhere to treatment. There is, however, a lack of information about what high-school learners know about TB symptoms, transmission, prevention, and their intention to seek healthcare regarding TB.

Improving health communication is an important step towards achieving changes in health behaviour, health-seeking behaviour, and social change (9, 10). Many theories have been developed and there has been a move away from models that focus on an individual to more inclusive models that include social networks, community, and societal aspects (9, 11, 12). For an infectious disease such as TB it is important that people understand transmission and the fact that TB can be treated and cured. The TB epidemic in South African communities has accompanied the HIV and AIDS epidemic, and there is much confusion about the presentation and progress of these different diseases (13). There is thus an urgent need to provide communities with the information that will enable them to prevent TB transmission and if symptomatic, to seek early treatment. A feasible approach to getting this information to communities is to reach households through their children, with an initial focus on older children of high-school age.

As part of a larger study which aimed to improve health literacy on TB, HIV, and sexually transmitted infections (STIs) among high-school learners in KwaZulu-Natal (KZN), South Africa, this article reports on factors associated with a positive intention to seek healthcare for TB treatment among high-school learners.

## Methods

This cross-sectional observational study was conducted in two districts of KZN among grade 9, 10, 11 learners (*n*=1,137) from 10 high schools (stratified by district) in 2008. Ugu (population 710,000) (14), a predominantly rural district in the southern part of the province with the population living in scattered homesteads; and the metropolitan area of eThekwini, with an estimated population of more than 3 million; were the chosen study sites (15). One class of grade 9, 10, and 11 learners in each school was randomly chosen for participation in the study with all learners invited to participate.

### Data collection

Data were collected using a self-administered questionnaire which was developed following 12 focus group discussions on TB, HIV, and STIs with learners (*n*=80) from schools in the rural (*n*=4) and urban (*n*=4) study sites. The questionnaire was piloted among the learners and edited to ensure clarity of the questions. These schools were not included in the main study. In addition to demographic details, the questionnaire surveyed learners’ knowledge about the mode of TB transmission (5 questions), TB symptoms (4 questions), interventions to prevent TB (5 questions), and knowledge as to whether TB is treatable, curable, and preventable (3 questions). They were also asked about having had TB themselves or having had a family member with TB. Learners were asked about their intention to take a family member for TB treatment, or to seek TB treatment themselves should the family member or they themselves, have symptoms, and their intention to ensure TB treatment adherence by either a family member or themselves, should they have TB. All questions were closed questions. Questions on their own or their family members’ experience of having TB; whether TB is treatable, curable, and preventable or not; and TB transmission; had three options (yes, no, and unsure). Questions on TB symptoms, interventions to prevent TB, intention to seek TB treatment, and ensure TB treatment adherence, had five options with 1 being strongly disagree and 5 being strongly agree. The questionnaires were translated into isiZulu and back-translated to ensure accuracy in English and isiZulu versions.

Two teams, each comprising a male and female young research assistant, were trained to collect the data. At each school, after obtaining written parental informed consent, and written assent from the learners, they explained the purpose of the study. They emphasised the importance of valid responses and that the questionnaires were anonymous. They then handed out the questionnaires to the learners for self-completion and explained that they should answer each question. On completion, the learners placed their questionnaires in envelopes which they then sealed. This process took about an hour.

### Data analysis

Data were entered into Epidata and analysed using IBM SPSS version 19 (SPSS Inc., Chicago, IL). Medians with ranges and frequencies were reported for continuous and categorical variables, respectively. The Cronbach's alpha for the elements on TB symptoms, transmission, and prevention ranged from 0.72 to 0.79. The independent variables were categorised as having correct knowledge or not. Participants who were unsure were classified as having incorrect knowledge. The four dependent variables were categorised as follows: if learners chose options 1 to 3 (strongly disagree, disagree, and unsure) it was categorised as the learner having no intention to seek TB treatment or adhere to TB treatment for themselves or their family; whereas options 4 (agree) and 5 (strongly agree) were categorised as having an intention to seek TB treatment or adhere to TB treatment for themselves or their family. Chi square was used to test for associations between independent categorical variables and the dependent variables under study. Independent variables which were significant on bivariate analysis were included in the multivariate analysis. The complex sample procedure in SPSS was used to correct for the nesting of students within schools. Logistic regression models testing for significant associations between the independent variables and the dependent variables under study, while controlling for age, sex, grade, and school location were explored. The accepted level of significance was 0.05 (*α*=0.05).

Ethical approval for the study was obtained from the University of KwaZulu-Natal's Biomedical Research Ethics Committee (BREC no: 038/08).

## Results

In total, 1,137 learners participated in the study. Information on one or more variables was missing for 23 learners who were excluded from the final analysis. Among the 1,114 learners, the overall median age was 17 years (range: 11–25 years). Females constituted 53% (*n*=590) of the participants (Table 1). Exploring associations between sex and knowledge, females (*n*=387, 65.6%) were significantly more likely than males (*n*=268, 51.1%) (*p*<0.01) to correctly answer that sharing utensils and clothing with a person who has TB did not transmit TB; and that one does not get TB by sharing a toilet with a person who has TB (females: *n*=485, 78.8%; males: *n*=352, 67.2%) (*p*<0.01). Females (*n*=426; 72.2%) were more likely to know that coughing blood is a TB symptom compared to males (*n*=346, 66.0%) (*p*=0.03). Interestingly, significantly more males (*n*=258, 49.2%) than females (*n*=237, 40.2%) (*p*<0.01) knew that coughing in an open space prevents TB spread. Significantly more females (*n*=511, 86.6%) than males (*n*=426, 81.3%) (*p*=0.02) knew that TB was treatable; had a family member on TB treatment (females: *n*=241, 61.8%; males: *n*=149, 38.2%) (*p*<0.01); and knew a person with TB who had been cured (females: *n*=335, 61.0%; males: *n*=214, 39.0%) (*p*<0.01).

**Table 1:** Demographic profile of high-school learners in KwaZulu-Natal, by area (*N*=1,114)

Characteristic	Urban (*n*=547)	Rural (*n*=567)	Total (*N*=1,114)
Age in years (median; range)	16 (13–23)	17 (11–25)	17 (11–25)
			
Sex (*n*, %)			
Male	260 (47.5)	264 (46.6)	524 (47.0)
Female	287 (52.5)	303 (54.4)	590 (53.0)
			
Grade (*n*, %)			
9	212 (38.8)	199 (35.1)	411 (36.9)
10	176 (32.2)	187 (33.0)	363 (32.6)
11	159 (29.1)	181 (31.9)	340 (30.5)
			
Living with a least one parent (*n*, %)			
Yes	375 (68.6)	439 (77.4)^a^	814 (73.1)
No	172 (31.4)	128 (22.6)	300 (26.9)

Chi square (*α*=0.05)

a <0.01.

Significantly more rural learners (*n*=439, 77.4%) lived with at least one parent compared to urban learners (*n*=375, 68.6%) (*p*=0.001) (Table 1). Significantly more urban learners (*n*=346, 63.3%) knew that you do not get TB from sharing utensils and clothing with a person who has TB as opposed to rural learners (*n*=309, 54.5%) (*p*=0.003). Significantly more urban learners (*n*=464, 84%) knew that a TB patient who is coughing can transmit TB as opposed to rural learners (*n*=442, 78.0%) (*p*=0.003). Significantly more rural learners (*n*=512; 90.3%) knew correctly that a person with TB should cover their mouth when they cough, compared to urban learners (*n*=469, 85.7%) (*p*=0.02).

More urban learners knew that TB is treatable (*n*=479, 87.6%) and curable (*n*=354, 64.7%) as compared to rural learners (TB treatable, *n*=458, 80.8%; TB curable, *n*=294, 51.9%) (TB treatable, *p*=0.002; TB curable, *p*<0.001). Significantly more urban learners (*n*=211, 38.6%) have ever had a family member on TB treatment compared to rural learners (*n*=179, 31.6%) (*p*=0.02). Furthermore, urban learners (*n*=305, 55.8%) knew a person who had been cured of TB compared to rural learners (*n*=244, 43.0%) (*p*<0.001). Positive intentions to take TB medication if infected with TB were reported significantly more among urban learners (*n*=486, 88.8%) than rural learners (*n*=464, 81.8%) (*p*=0.001) (Table 2).

**Table 2:** TB knowledge and health-seeking behaviour intentions among high-school learners in KwaZulu-Natal, by area (*N*=1,114)

	Urban (*n*=547)	Rural (*n*=567)	Total (*N*=1,114)
	
Characteristic	*n* (%)	*n* (%)	*n* (%)
TB transmission			
Get TB by using the same utensils and clothing as person with TB (no)^b^	346 (63.3)	309 (54.5)	665 (58.8)
Get TB by using the same toilet as person with TB (no)	410 (75.0)	407 (71.8)	817 (73.3)
Get TB if a person with TB coughs (yes)^b^	464 (81.8)	442 (78.0)	906 (81.3)
Get TB through witchcraft (no)	420 (76.8)	440 (77.6)	860 (77.2)
Get TB by having sex (no)	278 (50.8)	288 (50.8)	566 (50.8)
			
TB symptoms			
Loss of weight (yes)	328 (60.0)	327 (57.7)	665 (58.8)
Coughing for more than 3 weeks (yes)	352 (64.4)	350 (61.7)	702 (63.0)
Coughing blood (yes)	379 (69.3)	393 (69.3)	772 (69.3)
Night sweats (yes)	352 (64.4)	353 (62.3)	705 (63.3)
			
TB prevention			
When a person with TB coughs they should cover their mouth (yes)^a^	469 (85.7)	512 (90.3)	981 (88.1)
When a person with TB coughs they should go to an open space (yes)	229 (41.9)	266 (46.9)	495 (44.4)
When a person with TB coughs they should have good air circulation in the room (yes)	299 (54.7)	332 (58.6)	631 (56.6)
When a person with TB coughs they should cough in a closed room with other people in it (no)	440 (80.4)	451 (79.5)	891 (80.0)
When a person with TB coughs they should drink water and it will go away (no)	481 (87.9)	493 (84.9)	974 (87.4)
			
TB treatable/preventable			
TB is treatable (yes)^b^	479 (87.6)	458 (80.8)	937 (84.1)
TB is curable (yes)^b^	354 (64.7)	294 (51.9)	648 (58.2)
TB is preventable (yes)	313 (57.62)	314 (55.4)	627 (56.3)
			
Personal/family experience of TB			
Has ever had a family member on TB treatment (yes)^b^	211 (38.6)	179 (31.6)	390 (35.0)
Knows a person who has been cured of TB (yes)^b^	305 (55.8)	244 (43.0)	549 (49.3)
Have you been on TB treatment (yes)	29 (5.2)	37 (6.5)	66 (5.9)
Health-seeking behaviour			
If my family member has TB symptoms I will take them to a health facility (yes)	501 (91.6)	514 (90.7)	1,015 (91.1)
I will encourage them to take their TB treatment (yes)	519 (94.9)	530 (93.5)	1,049 (94.2)
If I cough for more than 3 weeks I will get tested for TB (yes)	431 (78.8)	430 (75.8)	861 (77.3)
I will take my TB medication (yes)^b^	486 (88.8)	464 (81.8)	950 (85.3)

TB, tuberculosis.

Chi square (*α*=0.05)

a <0.05;

b <0.01.

In multivariate analysis, certain variables which were significant in bivariate analysis (Table 3) remained significant when controlling for age, sex, grade, and school location. Learners who knew that TB is transmitted when a person with TB coughs were significantly more likely to intend to take their family members with TB symptoms to a health facility (OR: 1.56, 95% CI: 1.23–1.98). Learners who knew that coughing for more than 3 weeks (OR: 2.33, 95% CI: 1.35–4.00) and night sweats (OR: 3.12, 95% CI: 1.80–5.41) were TB symptoms were significantly more likely to intend to take a family member to a health facility if they had TB symptoms. Similarly, learners who knew that a person with TB coughing in a closed room where there were other people was an incorrect practice in preventing TB spread were significantly more likely to intend to take a family member to a health facility if they had TB symptoms (OR: 1.69, 95% CI: 1.05–2.75).

**Table 3:** Crude associations of TB knowledge with health-seeking behavioural intentions amongst high-school learners in KwaZulu-Natal [odds ratios (95% CI)], (*N*=1,114)

	Health-seeking behavioural intentions			
	
Characteristic	If my family member has TB symptoms I will take them to a health facility	I will encourage them to take their TB treatment	If I cough for more than 3 weeks I will get tested for TB	I will take my TB medication
Demographics	OR (95% CI)	OR (95% CI)	OR (95% CI)	OR (95% CI)
Age (≥17)	0.63 (0.41–0.96)	0.77 (0.46–1.28)	0.98 (0.75–1.31)	0.77 (0.55–1.07)
Sex (female)	1.52 (1.00–2.31)	1.63 (0.98–2.71)	1.20 (0.91–1.59)	0.89 (0.64–1.24)
Grade 9	Ref	Ref	Ref	Ref
Grade 10	1.21 (0.75–1.95)	0.98 (0.57–1.70)	0.98 (0.71–1.35)	1.75 (1.18–2.61)
Grade 11	1.57 (0.94–2.61)	2.51 (1.20–5.22)	2.04 (1.41–2.96)	1.81 (1.20–2.73)
Residence (urban)	1.12 (0.74–1.69)	1.29 (0.78–2.15)	1.18 (0.89–1.57)	1.77 (1.26–2.49)
				
TB transmission				
Get TB by using the same utensils and clothing as person with TB (no)	1.17 (0.78–1.77)	0.83 (0.49–1.39)	0.95 (0.71–1.27)	1.01 (0.72–1.42)
Get TB by using the same toilet as person with TB (no)	1.42 (0.92–2.21)	1.15 (0.66–1.99)	1.48 (1.09–2.00)	1.09 (0.75–1.57)
Get TB if a person with TB coughs (yes)	2.40 (1.53–3.76)	2.56 (1.50–4.36)	1.17 (0.82–1.67)	1.63 (1.10–2.39)
Get TB through witchcraft (no)	0.96 (0.59–1.58)	1.02 (0.56–1.84)	1.45 (1.05–1.99)	0.98 (0.66–1.46)
Get TB by having sex (no)	1.01 (0.67–1.53)	1.07 (0.65–1.77)	1.05 (0.79–1.39)	0.93 (0.67–1.29)
				
TB symptoms				
Loss of weight (yes)	2.87 (1.87–4.42)	2.77 (1.64–4.67)	2.60 (1.95–3.47)	2.57 (1.83–3.61)
Coughing for more than 3 weeks (yes)	4.07 (2.62–6.32)	4.89 (2.80–8.55)	2.22 (1.67–2.95)	3.01 (2.14–4.23)
Coughing blood (yes)	3.17 (2.09–4.82)	4.56 (2.69–7.71)	2.96 (2.22–3.96)	2.82 (2.01–3.95)
Night sweats (yes)	5.08 (3.22–8.02)	4.58 (2.64–7.93)	2.16 (1.62–2.87)	2.41 (1.72–3.37)
				
TB prevention				
When a person with TB coughs they should cover their mouth (yes)	1.74 (1.01–3.00)	2.15 (1.15–3.99)	1.43 (0.95–2.14)	1.75 (1.11–2.74)
When a person with TB coughs they should go to an open space (yes)	0.87 (0.58–1.32)	1.13 (0.68–1.88)	1.14 (0.86–1.52)	0.91 (0.66–1.27)
When a person with TB coughs they should have good air circulation in the room (yes)	0.92 (0.60–1.39)	1.37 (0.83–2.27)	1.01 (0.76–1.34)	1.03 (0.74–1.43)
When a person with TB coughs they should cough in a closed room with other people in it (no)	2.17 (1.39–3.39)	2.00 (1.16–3.44)	1.41 (1.01–1.97)	1.70 (1.17–2.48)
When a person with TB coughs they should drink water and it will go away (no)	1.39 (0.79–2.44)	1.82 (0.96–3.43)	1.11 (0.73–1.67)	1.62 (1.04–2.54)
				
TB treatable/preventable				
TB is treatable (yes)	1.29 (0.76–2.44)	0.84 (0.41–1.74)	1.24 (0.86–1.79)	1.54 (1.02–2.34)
TB is curable (yes)	1.18 (0.78–1.78)	0.92 (0.55–1.54)	1.14 (0.86–1.51)	1.31 (0.94–1.83)
TB is preventable (yes)	0.59 (0.38–0.91)	0.97 (0.59–1.61)	1.32 (0.99–1.75)	0.98 (0.70–1.37)
				
Personal/family experience of TB				
Has ever had a family member on TB treatment (yes)	1.14 (0.73–1.77)	1.14 (0.67–1.94)	1.22 (0.90–1.64)	1.27 (0.89–1.82)
Knows a person who has been cured of TB (yes)	1.55 (1.02–2.37)	1.14 (0.69–1.89)	1.44 (1.09–1.91)	1.37 (0.97–1.91)
Have you been on TB treatment yourself (yes)	0.97 (0.41–2.32)	2.05 (0.49–8.56)	1.09 (0.59–2.02)	0.97 (0.48–1.93)

TB, tuberculosis.

Learners who knew that coughing for more than 3 weeks (OR: 2.69, 95% CI: 1.19–6.09), coughing blood (OR: 2.24, 95% CI: 1.33–3.76), and night sweats (OR: 2.25, 95% CI: 1.09–4.64) were TB symptoms were significantly more likely to intend to encourage a family member to adhere to their TB treatment (Table 4).

**Table 4:** Adjusted associations of TB knowledge with health-seeking behavioural intentions amongst high-school learners in KwaZulu-Natal [odds ratios (95% CI)], (*N*=1,114)

	Health-seeking behavioural intentions			
	
Characteristic	If my family member has TB symptoms I will take them to a health facility	I will encourage them to take their TB treatment	If I cough for more than 3 weeks I will get tested for TB	I will take my TB medication
Demographics	OR (95% CI)	OR (95% CI)	OR (95% CI)	OR (95% CI)
Age (≥17)	0.62 (0.32–1.19)	0.77 (0.32–1.86)	0.89 (0.63–1.27)	0.61 (0.33–1.13)
Sex (female)	1.27 (0.82–1.96)	1.51 (0.78–2.91)	1.07 (0.84–1.37)	0.69 (0.45–1.05)
Grade 9	Ref	Ref	Ref	Ref
Grade 10	1.32 (0.69–2.50)	0.96 (0.41–2.26)	1.01 (0.56–1.82)	2.07 (0.98–4.36)
Grade 11	1.40 (0.59–3.36)	2.16 (0.72–6.45)	1.81 (1.12–2.92)	1.77 (0.83–3.79)
Residence (urban)	1.02 (0.67–1.57)	01.21 (0.84–1.19)	1.16 (0.59–2.25)	1.80 (0.88–3.69)
				
TB transmission				
Get TB by using the same toilet as person with TB (no)			1.39 (0.99–1.94)	
Get TB if a person with TB coughs (yes)	1.56 (1.23–1.98)	1.51 (0.82–2.79)		1.01 (0.45–2.30)
Get TB through witchcraft (no)			1.20 (0.91–1.59)	
				
TB symptoms				
Loss of weight (yes)	1.49 (0.83–2.70)	1.35 (0.69–2.60)	1.85 (1.38–2.49)	1.71 (0.98–3.00)
Coughing for more than 3 weeks (yes)	2.33 (1.35–4.00)	2.69 (1.19–6.09)	1.47 (1.05–2.07)	2.04 (1.45–2.87)
Coughing blood (yes)	1.42 (0.87–2.31)	2.24 (1.33–3.76)	2.08 (1.44–3.01)	1.81 (1.24–2.62)
Night sweats (yes)	3.12 (1.80–5.41)	2.25 (1.09–4.64)	1.26 (0.97–1.65)	1.42 (0.80–2.53)
				
TB prevention				
When a person with TB coughs they should cover their mouth (yes)	1.29 (0.68–2.49)	1.61 (0.65–4.04)		1.62 (0.99–2.64)
When a person with TB coughs they should cough in a closed room with other people in it (no)	1.69 (1.05–2.75)	1.48 (0.62–3.49)	1.16 (0.62–2.16)	1.23 (0.83–1.82)
When a person with TB coughs they should drink water and it will go away (no)				1.42 (0.91–2.21)
				
TB treatable/preventable				
TB is treatable (yes)				1.21 (0.61–2.41)
				
Personal/family experience of TB				
Knows a person who has been cured of TB (yes)	1.16 (0.67–2.01)		1.09 (0.79–1.49)	

TB, tuberculosis.

Grade 11 learners (OR: 1.81, 95% CI: 1.12–2.92) and learners who knew that coughing for more than 3 weeks (OR: 1.47, 95% CI: 1.05–2.07), coughing blood (OR: 2.08; 95% CI:1.44–3.01), and weight loss (OR: 1.85; 95% CI: 1.38–2.49) were TB symptoms were significantly more likely to indicate that they would get tested for TB, if they coughed for more than 3 weeks. Learners who knew that coughing for more than 3 weeks (OR: 2.04; 95% CI: 1.45–2.87) and coughing blood (OR: 1.81; 95% CI: 1.24–2.62) were TB symptoms were significantly more likely to indicate that they would take their TB treatment should they have TB (Table 4).

## Discussion

Although this study was a cross-sectional design limiting conclusions about the direction of the associations, its findings provide important insight into knowledge and the intention to seek healthcare among high-school learners in a country with a TB epidemic. There was a strong association between knowledge of TB symptoms, transmission, prevention, and positive health-seeking behavioural intentions for themselves and family members.

This study reflected an awareness about TB transmission, and whether TB is treatable and curable, in addition to a readiness to take TB medication among urban learners. The inequalities among urban–rural dwellers with respect to TB knowledge and practices have been reported on previously in developing countries with TB epidemics (16, 17). It is well established that there is inequity in the provision of health services between urban and rural areas in South Africa (18). The access for people to TB services and health information is likely to be better in the urban areas as opposed to rural areas. Lack of access to TB services and health information in rural areas could account for the inequalities in TB knowledge and practices found in this study.

In studies among adults, it has been shown that females are more likely to delay seeking treatment for TB (19, 20). Despite finding significant associations between sex and knowledge on TB transmission, symptoms, and disease on bivariate analysis, there was no association between sex and intention to seek TB treatment or to adhere to TB treatment in our study. This is a positive finding in these high-school learners in that gender is not influencing their health-seeking behaviour. In South Africa to date, males are often less likely to seek and receive treatment than females (21). In addition, age did not influence potential health-seeking behaviour as no significant associations were found in our study.

Urban learners were twice as likely to intend taking their TB medication as opposed to rural learners, which is a concern since 38% of South Africa's population resides in rural areas (22). This would suggest that TB awareness campaigns need to target rural areas so as to encourage health-seeking behaviours.

Learners in the higher grades were more likely to intend seeking TB treatment and intended to be compliant with TB treatment. Literacy does influence health-seeking behaviour and studies have shown that the more literate patient is likely to seek TB treatment (23, 24). Thus, strategies need to be developed to transfer TB knowledge to the learners in lower grades. Peer mentorship programmes on other health aspects have been used where older students mentor younger students at school (25). This is one possible method that can be used to raise awareness among younger learners with respect to TB.

Educational programmes on oral health and HIV/AIDS have been introduced into school curricula in China with considerable success (25, 26). Educational programmes for raising awareness among learners on TB in our study population need to examine ways in which learners’ TB knowledge can be translated into positive health-seeking behaviour. Clearly, based on responses, some learners had experiences of TB because of the epidemic. Their experiences and knowledge should be integrated into education programmes focusing on raising TB awareness among learners. ‘Life skills’ is a subject taught at schools throughout South Africa (27). Content on TB and other similar diseases can be integrated into the ‘life skills’ curriculum which will serve to increase awareness among learners. Health literacy about TB may help to reduce the stigma associated with the disease and additional information provided for high-school learners may reduce their risk of infection and delayed treatment. Learners who understand the importance of early diagnosis for TB and adherence to TB treatment can explain to other family members the rationale for such treatment and the benefits for the individual, his or her family, and the community.

We measured the intentions of learners to implement health-seeking practices. Wiedemann et al. showed that although intention does have a positive effect on behaviour, planning is a mediator of the intention–behaviour relationship (28). Hence, as part of the learning intervention, learners should be taught to plan, so as to translate intentions into positive health-seeking behaviour. In addition, a study on behavioural intention to adhere to HIV care has shown that knowledge and intention were associated with viral suppression which would suggest that in our study learners’ knowledge of TB symptoms, transmission, prevention, and association with the intention to seek TB treatment is likely to translate into positive health-seeking behaviour (29).

## Conclusion and recommendation

TB is a preventable disease and early diagnosis is important in order to receive treatment and reduce transmission of the infection. The severe epidemic in South Africa requires that learners’ awareness about TB increases and that they recognise the symptoms of TB and improve their health-seeking behaviour. Educational programmes on TB focused at high-school learners can improve their knowledge of TB. These high-school learners can also act as a conduit to other family members in recognising symptoms, knowing how to reduce transmission through improved hygienic practices, and encouraging them to visit the health facilities for treatment and encouraging compliance.

## References

[1] Wood R, Lawn SD, Johnstone-Robertson S, Bekker LG (2011). Tuberculosis control has failed in South Africa – time to reappraise strategy. S Afr Med J.

[2] Calver AD, Falmer AA, Murray M, Strauss OJ, Streicher EM, Hanekom M (2010). Emergence of increased resistance and extensively drug-restant tuberculosis despite treatment adherence, South Africa. Emerg Infect Dis.

[3] National Department of Health, Republic of South Africa (2009). National TB Management Guidelines 2009.

[4] Middelkoop K, Bekker LG, Myer L, Dawson R, Wood R (2008). Rates of tuberculosis transmission to children and adolescents in a community with a high prevalence of HIV infection among adults. Clin Infect Dis.

[5] Mahomed H, Ehrlich R, Hawkridge T, Hatherill M, Geiter L, Kafaar F (2013). TB incidence in an adolescent cohort in South Africa. PLoS One.

[6] Department of Basic Education, Republic of South Africa South African Country Report: progress on the implementation of the regional education and training plan. http://www.education.gov.za/LinkClick.aspx?fileticket=B3Ql1NJKS3g%3D&t.

[7] Fortenberry JD, McFarlane MM, Hennessy M, Bull SS, Grimley DM, St. Lawrence J (2001). Relation of health literacy to gonorrhoea related care. Sex Transm Infect.

[8] Jensen PA, Lambert LA, Iademarco MF, Ridzon R (2005). Guidelines for preventing the transmission of Mycobacterium tuberculosis in health-care settings. MMWR Recomm Rep.

[9] Dutta-Bergman MJ (2005). Theory and practice in health communication campaigns: a critical interrogation. Health Commun.

[10] Nutbeam D (2000). Health literacy as a public health goal: a challenge for contemporary health education and communication strategies into the 21st century [serial on internet]. http://heapro.oxfordjournals.org/content/15/3/259.full.pdf.

[11] McLeroy KR, Bibeau D, Steckler A, Glanz K (1988). An ecological perspective on health promotion programs. Health Educ Q.

[12] Ryan P (2009). Integrated theory of health behavior change: background and intervention development. Clin Nurse Spec.

[13] Murombedzi C TB: seek treatment early. http://www.herald.co.zw/tb-seek-treatment-early/.

[14] Ugu District Municipality Integrated Development Plan Review (IDP). 2011/2012. Ugu District Municipality. http://devplan.kzntl.gov.za/idp_reviewed_2011_12/IDPS/DC21/Adopted/UGU%20District%20%20IDP%20Final%20%202011-%202012pdf.

[15] eThekwini Municipality Medium Term Expenditure Framework (MTEF): 2011/12 to 2013/14. eThekwini Municipality. http://www.durban.gov.za/durban/government/treasury/budget/Medium%20Term%20Revenue%20and%20Expenditure%20Framework%202011.2012-new.pdf.

[16] Singh UP, Bala A, Goel RKD (2006). Knowledge about tuberculosis in senior school students of Punjab. Indian J Comm Med.

[17] Mushtaq MU, Shahid U, Abdullah HM, Saeed A, Omer F, Shad MA (2011). Urban-rural inequities in knowledge, attitudes and practices regarding tuberculosis in two districts of Pakistan's Punjab province. Int J Equity Health.

[18] Silal SP, Penn-Kekana L, Harris B, Birch S, McIntyre D (2012). Exploring inequalities in access to and use of maternal health services in South Africa. BMC Health Serv Res.

[19] Huong NT, Vree M, Duong BD, Khanh VT, Loan VT, Co NV (2007). Delays in the diagnosis and treatment of tuberculosis patients in Vietnam: a cross-sectional study. BMC Public Health.

[20] Storla DG, Yimer S, Bjune GA (2008). A systematic review of delay in the diagnosis and treatment of tuberculosis. BMC Public Health.

[21] Skordis-Worralla J, Hanson K, Mills A (2011). Estimating the demand for health services in four poor districts of Cape Town, South Africa. Int Health.

[22] Phaswana-Mafuya N (2012). Ageing and health: the SAGE survey. http://www.phasa.org.za/wp-content/uploads/2012/05/Nancy_Aging-and-Health_article.pdf.

[23] Date J, Okita K (2005). Gender and literacy: factors related to diagnostic delay and unsuccessful treatment of tuberculosis in the mountainous area of Yemen. Int J Tuberc Lung Dis.

[24] Karcher MJ (2005). The effects of developmental mentoring and high school mentors’ attendance on their younger mentees’ self-esteem, social skills and connectedness. Psychol Schools.

[25] Petersen PE, Peng B, Tai B, Bian Z, Fan M (2004). Effect of a school-based oral health education programme in Wuhan City, Peoples Republic of China. Int Dent J.

[26] Gao X, Wu Y, Zhang Y, Zhang N, Tang J, Qiu J (2012). Effectiveness of school-based education on HIV/AIDS knowledge, attitude, and behavior among secondary school students in Wuhan, China. PLoS One.

[27] Visser MJ (2005). Life skills training as HIV/AIDS preventive strategy in secondary schools: evaluation of a large- scale implementation process. SAHARA J.

[28] Wiedemann AU, Schüz B, Sniehotta F, Scholz U, Schwarzer R (2009). Disentangling the relation between intentions, planning, and behaviour: a moderated mediation analysis. Psychol Health.

[29] Nelsen A, Trautner BW, Petersen NJ, Gupta S, Rodriguez-Barradas M, Giordano TP (2012). Development and validation of a measure for intention to adhere to HIV treatment. AIDS Patient Care STDS.

